# Cognitive rehabilitation interventions after stroke: protocol for a systematic review and meta-analysis of randomized controlled trials

**DOI:** 10.1186/s13643-021-01607-7

**Published:** 2021-03-04

**Authors:** Qing Zhao, Xue Wang, Tao Wang, Adam A. Dmytriw, Xiao Zhang, Kun Yang, Jichang Luo, Xuesong Bai, Nan Jiang, Bin Yang, Yan Ma, Liqun Jiao, Yunyan Xie

**Affiliations:** 1 Department of Neurology, Xuanwu Hospital, Capital Medical University, No. 45 Changchun Street, Beijing, 100053 China; 2 Department of Clinical Medicine, Peking Union Medical College, No. 5 Dongdan Three Street, Beijing, China; 3 Medical Library, Xuanwu Hospital, Capital Medical University, No. 45 Changchun Street, Beijing, China; 4 Department of Neurosurgery, Xuanwu Hospital, Capital Medical University, No. 45 Changchun Street, Beijing, China; 5 China International Neuroscience Institute (China-INI), No. 45 Changchun Street, Beijing, China; 6 Department of Radiology, Brigham and Women’s Hospital, Harvard Medical School, 75 Francis St, Boston, MA USA; 7 Department of Evidence-Based Medicine, Xuanwu Hospital, Capital Medical University, No. 45 Changchun Street, Beijing, China; 8 Department of Interventional Neuroradiology, Xuanwu Hospital, Capital Medical University, No. 45 Changchun Street, Beijing, China

**Keywords:** Stroke, Cognitive impairment, Cognitive rehabilitation

## Abstract

**Background:**

Stroke is the second leading cause of death worldwide, and 53.4% of stroke survivors suffer from post-stroke cognitive impairment. Post-stroke cognitive impairment can increase hospitalization rate and cost of care and decrease the quality of life of stroke patients. To date, multiple cognitive rehabilitation interventions have been tested in stroke populations with post-stroke cognitive impairment. However, the most efficacious intervention has not been established. This systematic review aims to compare the efficacy of cognitive rehabilitation interventions for patients with post-stroke cognitive impairment.

**Methods:**

We will search MEDLINE, EMBASE, CENTRAL, PsycINFO, CINAHL, PubMed, and clinical trial registries to identify eligible randomized clinical trials with no restrictions in the date of publication and language. Studies conducted with patients aged 18 or over, with the presence of cognitive impairment after being diagnosed with stroke will be included. Studies will be restricted to randomized controlled trials comparing a cognitive rehabilitation intervention with another intervention. The primary outcome is any clinical changes in the general or specific cognitive domain (e.g., executive function, attention, memory, or perception). The secondary outcomes that will be collected include adverse effects (e.g., stroke, disability, or mortality) and quality of life. Two independent reviewers will assess articles to identify trials eligible for inclusion. Data extraction and risk of bias assessment of the included studies will also be done independently. Any discrepancies will be solved by discussion, or a third reviewer will be consulted if necessary. A meta-analysis will be carried out if appropriate.

**Discussion:**

This systematic review for patients with post-stroke cognitive impairment will assess the efficacy of cognitive rehabilitation interventions. And our results will help clinical decision-making and support the development of clinical practice guidelines.

**Trial registration:**

**Systematic review registration:** PROSPERO CRD42020173988

**Supplementary Information:**

The online version contains supplementary material available at 10.1186/s13643-021-01607-7.

## Background

### Description of the condition

Stroke is the second leading cause of death and the third leading cause of disability worldwide [1], and stroke survivors commonly develop post-stroke cognitive impairment (PSCI). Cognition is composed of multiple domains, including attention, memory, executive function, visuospatial ability, verbal information, language, and other aspects. Patients with PSCI may have damage to one or more cognitive domains, and the current thinking is that stroke tends to impact more deleteriously on attention and executive function compared with its impact on memory [2]. A recent study showed that averaged performance of stroke survivors in three specific cognitive domains: action speed, executive functions, and language, might be the optimal criterion for PSCI [3]. A recent systematic review and meta-analysis has identified that the prevalence of PSCI is 53.4%, measured within 1.5 years post-stroke [4]. However, PSCI also has persistent pervasiveness. The prevalence measured at 5 years and 14 years is 22% and 21%, respectively [5]. Two partially conflicting hypotheses were proposed to reveal the mechanism linking stroke to PSCI [6]. The first hypothesis highlights that stroke itself is a central factor in the development of cognitive impairment, and therefore, performing optimal acute stroke care and preventing stroke recurrence might be the most effective therapy for PSCI. A study found that severity, subtype, and location of the stroke; the volume of infarct; and recurrent stroke were all significantly associated with PSCI, which might support this hypothesis [7]. The other hypothesis emphasizes that PSCI might take place because stroke aggravates multiple clinically silent vascular risk factors, such as hypertensive vasculopathy or cerebral amyloid angiopathy. PSCI can increase the institutionalization rate [8], and costs of care [9], while decreasing the quality of life [10]. Currently, cognitive impairment is determined using multiple neurophysiological yardsticks such as the Mini-Mental State Examination (MMSE) and Montreal Cognitive Assessment (MoCA); there are also neurophysiological tests to examine the impairment on a single cognitive domain, like executive function and memor y[11].

### Description of the intervention

**Fig. 1:**
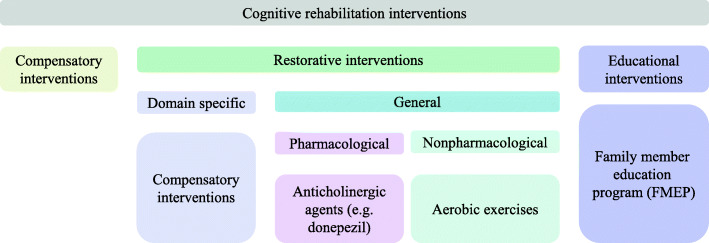
Cognitive rehabilitation interventions

On the basis of assessing and understanding the patients’ cognitive impairment, cognitive rehabilitation is a systematic therapeutic activity-oriented functionally [12]. Cognitive rehabilitation includes interventions that might be compensatory, educational, or restorative (see Fig. 1). Compensatory interventions tend to adapt to the external environment and improve the ability of patients to use aids and tools to overcome the impairment. One such example is the electronic paging system. Educational interventions, such as the family member education program, aim to help the patients and their family members to improve the understanding of stroke and PSCI, including definition, management, measurement, and metal support. Restorative interventions aim to directly restore the impaired function of patients with PSCI, including domain-specific interventions and interventions for generalized cognitive impairment. Generalized cognitive rehabilitation interventions include pharmacological and nonpharmacological interventions.

No definitive pharmacotherapies have been proven for recovery from PSCI, but some agents have the potential to be used in treating PSCI. Anticholinergic agents, such as donepezil, are common drugs used to treat Alzheimer’s disease and now show significant efficacy in the treatment of PSCI. The recommendation from the American Heart Association/American Stroke Association (AHA/ASA) has suggested that donepezil was effective for enhancing cognition in patients suffering from vascular cognitive impairment [13]. What is more, a recent meta-analysis found that anticholinergic agents can stably improve cognitive function without increasing the risk of side effects in patients with PSCI [14]. An epidemiological study has demonstrated that hypertension in middle age can increase the likelihood of cognitive impairment when getting older [15]. What is more, studies found that high blood pressure has an association with PSCI. A clinical trial with 3.9 years of follow-up found that antihypertensive agents (perindopril and indapamide) can reduce risks of cognitive decline, possibly by preventing recurrent stroke [16]. Further studies show that calcium channel blockers, renin-angiotensin system blockers, and other specific antihypertensive drugs may show particular benefit in the prevention of cognitive decline [17]. Another promising drug might be escitalopram, which is an antidepressant drug. A study has shown that escitalopram may improve global cognitive function, especially in verbal and visual memory functions, and the effect might be independent of the antidepressant effect [18]. Fluoxetine, another antidepressant drug, has been shown to enhance motor recovery in the FLAME trial [19]. In the FOCUS trial, fluoxetine demonstrated no benefit in improving functional outcomes after acute stroke, although reducing the occurrence of post-stroke depression [20]. Further studies are needed to confirm the effect of antihypertensive and antidepressant drugs on cognitive rehabilitation of PSCI.

Several nonpharmacological treatments have shown positive results on the rehabilitation of PSCI. Aerobic exercises are activities with highly automated movements, and adequate aerobic exercises can benefit the cardiovascular and respiratory function of the body. A systematic review that included trials assessing the influence of aerobic exercise on PSCI shows that the duration of aerobic exercises can improve global cognitive ability, as well as specific domains of cognition such as attention and memory [21]. Aerobic exercises in these trials include bicycle cycling, tai chi, yoga, and treadmill exercise. Swatridge et al. also found that moderate-intensity aerobic exercise can have acute effects on cognitive control [22]. In this study, researchers found that people with chronic stroke can process information more quickly and control attention better, finishing 20 min of aerobic exercise. And electroencephalography showed improved cortical processing performance during a cognitive task after the aerobic exercises.

Computer-assisted cognitive rehabilitation (CACR) is another highlighted cognitive rehabilitation intervention. Compared with the conventional cognitive rehabilitation method, which is paper/pencil exercise, CACR has many advantages: (1) it has the flexibility to adjust the cognitive training on the basis of each patient’s specific neuropsychological patterns so that the damaged location can be better stimulated [23]; (2) it can give instant feedback and shorten treatment time [24]; (3) by incorporating video games, stroke patients might have more motivation for therapy. The clinical use of CACR has increased, and amounts of studies show its efficacy in improving attention, memory, executive function, visuospatial neglect, and other cognitive declines [25–27]. However, some studies found very limited effects of CACR on working memory and no effects on cognitive function compared to the control group [28]. A randomized controlled trial in 2019 also found no effects of CACR on facilitating the achievement of functional memory goals [29]. Further replicates of these findings are needed to confirm whether CACR shows efficacy in cognitive rehabilitation.

Noninvasive brain stimulation (NIBS), including transcranial current stimulation and transcranial magnetic stimulation, can modulate the excitability of specific brain regions and their participated networks noninvasively, influencing sensorimotor and cognitive abilities. Currently, NIBS is a promising diagnostic and therapeutic measure. NIBS has been well documented in improving language function after stroke [30–32]. A 10-Hz repetitive transcranial magnetic stimulation (rTMS) has been shown to improve PSCI [33]. What is more, a recent overview of systematic reviews about rTMS in stroke patients shows evidence for cognitive rehabilitation in hemineglect [34]. Although the use of NIBS for cognitive rehabilitation in other cognitive domains remains largely uncertain, results from healthy systems suggest that NIBS is a promising modality to enhance other cognitive functional recoveries (e.g., memory) in stroke patients [35].

### How the intervention might work

Restorative cognitive rehabilitation interventions aim to improve the impaired brain functions in patients with PSCI. And interventions might show efficacy in cognitive rehabilitation via targeting lesions on neuroanatomical structures after stroke. One example is anticholinergic drugs. Using whole-hemisphere sections, Selden et al. discovered two highly organized and discrete bundles of cholinergic fibers, which extend from the nucleus basalis to the cerebral cortex and amygdala [36]. Localized strokes might interrupt these bundles and reduce acetylcholine activity, and the aim of anticholinergic agents is to partially improve the impaired Ach activity. Another example might be NIBS. Cognitive functions are attributable to dynamic interactions of brain areas instead of operations of a single brain area [37]. NIBS could transcranially modulate the excitability of brain regions and their participated networks, of which the function could be damaged by stroke. Studies have found that the dorsal lateral prefrontal cortex, cerebellum, and posterior parietal cortex are the potential targets for NIBS [35].

Improving cerebral perfusion might be another target for cognitive rehabilitation. The prevalence of cerebral microbleeds (CMBs) is about 34% in ischemic stroke patients and 60% in hemorrhagic stroke patients [38]. And the presence of CMBs independently associates with mild cognitive impairment in patients [39]. Study showed that hypertension is a risk factor for CMBs [40]. Besides, hypertension could disrupt cerebral autoregulation, reduce cerebral perfusion, and limit the ability of the brain to clear harmful proteins [41] and, therefore, may impair cognition. Antihypertensive therapy might benefit the cognitive decline by correcting these factors. Physical exercises, including aerobic exercise, has been shown to increase perfusion and plasticity, and affect synaptic structure and strength through inducing central and peripheral growth factor s[42]. Therefore, aerobic exercises might benefit cognition rehabilitation by combining their effects on neuroanatomical structures and vascular function.

### Why it is important to do this review?

Cognitive impairment is found in approximately half of the stroke survivors and brings a heavy burden to both the patients’ family and the public health system. Although amounts of cognitive rehabilitation interventions have been investigated, there is no consensus in the decision of the best rehabilitative intervention for PSCI, and the intervention type, optimal treatment intensity, and timing to ensure effectiveness need further exploration. To our knowledge, another systematic review compared the efficacy of cognitive rehabilitation interventions in treating PSCI, but it only included literature from 2009 through 2014 [43]. Several important clinical trials have been published since then [44–47]. As a result, an up-to-date review is needed. The conclusions of our study may substantially help to address uncertainty in practice and inform clinical decision-making.

## Objectives

The study will aim to compare the efficacy of cognitive rehabilitation interventions for patients with PSCI.

## Methods

This protocol is being reported according to the Preferred Reporting Items for Systematic Reviews and Meta-Analyses Protocol 2015 (PRISMA-P) checklist (see Additional file 1) [48]. This protocol has been registered on the PROSPERO database (CRD42020173988). Any revision of this protocol and the whole review process will be updated timely on the PROSPERO registration.

### Criteria for considering studies for this review

#### Types of studies

We will include all randomized controlled trials that compare different cognitive rehabilitation interventions in treating people with cognitive impairment after stroke. Studies published from inception in any language will be included.

#### Types of participants

For study patients, we will include adults (aged over 18) of either sex and any ethnicity, with the presence of cognitive impairment after being diagnosed with stroke. In this review, we will accept the diagnosis of cognitive impairment made by any validated neuropsychological tests, such as MMSE, MoCA, or other domain-specific cognitive tests. We will also accept the diagnosis of cognitive impairment made by experienced researchers.

#### Types of interventions

We will consider any pharmacological or nonpharmacological interventions delivered alone.

Pharmacological interventions may include:

- Anticholinergic therapy, e.g., donepezil.
- Antihypertensive, e.g., indapamide.
- Antiplatelet, e.g., dipyridamole.
- Antidepressants, e.g., escitalopram.

Nonpharmacological interventions may include:

- Conventional cognitive training
- Computer-assisted cognitive training
- Aerobic exercises
- Music therapy
- Virtual reality
- Noninvasive brain stimulation
- Acupuncture
- Educational therapy, e.g., intensive caregiver education program.

Comparison will be done among the interventions mentioned above.

#### Types of outcome measures

##### Primary outcomes

- Any clinical changes in the general or specific cognitive domain (e.g., executive function, attention, memory, or perception), measured by any validated measures, including but not limited to screening instruments such as MMSE and MoCA, and validated measure of domain-specific cognitive function such as Trail Making Tests A and B, as well as Stroop Test.

##### Secondary outcomes

- Adverse effects, e.g., stroke, disability, or mortality, as defined by the original researchers.
- Quality of life, measured by any validated measure.

## Search strategy

### Electronic searches

The following electronic databases for relevant studies will be searched, with no restrictions in publication date or language:

- Cochrane Central Register of Controlled Trials (CENTRAL), Cochrane Database of Systematic Reviews, and Cochrane Methodology Register in the Cochrane Library (latest issue)
- EMBASE (from 1974 to present)
- MEDLINE (from 1946 to present)
- PsycINFO (from 1887 to present)
- CINAHL (from 1974 to present)
- PubMed (from 1966 to present)

Also, we will also search the US National Institutes of Health Ongoing Trials Register ClinicalTrials.gov (www.clinicaltrials.gov/) for ongoing trials register. An experienced librarian has developed the detailed draft search strategy for the MEDLINE database (see Additional file 2) and then adapted it for searching studies in other databases. The search strategy has been revised by another experienced librarian.

### Searching other resources

We will also search the reference lists of included trials, systematic reviews, and meta-analyses identified during the screening process to identify other eligible trials. Grey literature, such as conference proceedings will also be searched.

## Data collection and analysis

### Selection of studies

**Fig. 2:**
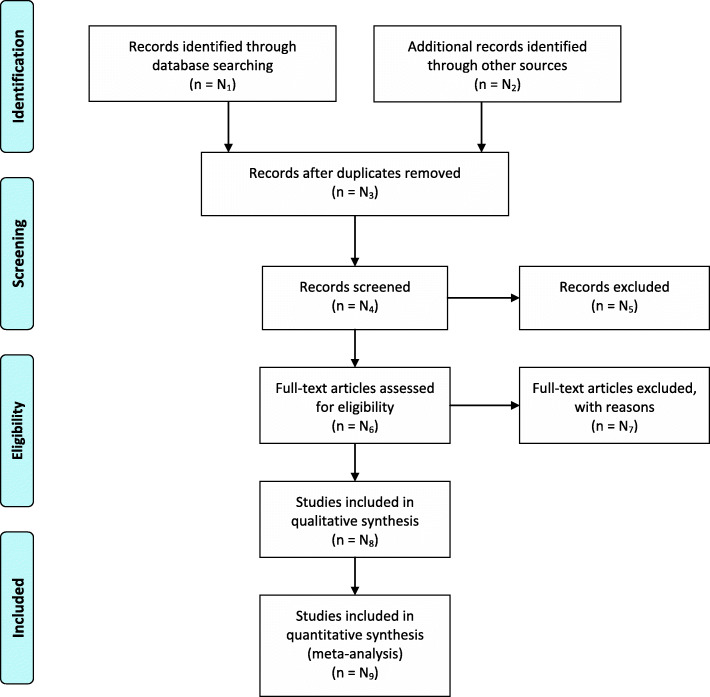
PRISMA 2009 Flow Diagram

We will store citations using the EndNote software (https://www.endnote.com/) with duplicates removed. The screening and selection will be completed in two levels. In level one, two review authors (JL and XW) will independently screen the titles and abstracts of every record from the list of results of our literature searching activity to identify all potentially relevant trials. In level two, the full text of all potentially relevant records from level one screening will be retrieved, and the same two reviewers will independently examine these and the reasons for excluding the ineligible trials will be recorded. We will calculate inter-rater reliability from a pilot study before each screening level using a predesigned test form (see Additional file 3) [49] and then launch the formal screening if the high agreement (≥ 80%) between two reviewers can be achieved. If discrepancies are found on study selection, the two review authors will further discuss, and a third review author (TW) will be consulted if necessary. The study selection process will be shown in a PRISMA flow diagram (see Fig. 2) [50].

### Data extraction and management

All study data will be recorded using a data extraction form created in Excel. Two review authors (JL and XW) will independently extract data from all included studies. The following detailed trial characteristics will be extracted: study characteristics (e.g., date of publication, study design, settings, country), characteristics of the patient (e.g., number enrolled in each group, age, gender, types of stroke, duration after stroke), interventions (e.g., types of interventions, comparison), outcome results (e.g., clinical changes in cognition, adverse effects, and specific measure of the quality of life). Similar to study selection, inter-rater reliability will also be calculated for conformation of high agreement (≥ 80%). Any disagreements on data extraction will be resolved through discussion, or by recourse to a third review author (TW).

### Assessment of risk of bias in included studies

The risk of bias for each included study will be assessed independently by two review authors (JL and XW), conforming to the criteria outlined in Chapter 8 of the *Cochrane Handbook for Systematic Reviews of Interventions* [51]. Any discrepancies on assessment will be resolved by further discussion or by involving a third review author (TW). The risk of bias in the following domains will be assessed:

- Random sequence generation
- Allocation concealment
- Blinding of participants and investigators
- Blinding of outcome assessment
- Incomplete outcome data
- Selective outcome report
- Other bias

We will grade the risk of bias of studies as low, high, or uncertain in each of these domains. Information from the study report will be provided with a justification for our judgment in the “Risk of bias” tables.

### Assessment of heterogeneity

We will measure heterogeneity among the trials in each analysis using the *I*^2^ statistic, and the heterogeneity will be considered as low (*I*^2^ = 0 to 40%), moderate (*I*^2^ = 40 to 70%), and substantial (*I*^2^ = 70 to 100%). If heterogeneity is found, we will explore the potential sources via subgroup analyses.

### Dealing with missing data

If we encounter missing data, the authors of the original trials and studies will be contacted to request access to further study data. When this is impossible, we will conduct an imputation approach using informative missing odds ratio (IMOR) for dichotomous data and informative missingness difference of means (IMDoM) for continuous data [52, 53]. And we will then conduct a sensitivity analysis to ensure no bias of the final results is created.

### Assessment of reporting biases

Published and unpublished studies will be searched comprehensively to minimize reporting biases. If we identify 10 or more studies, possible reporting biases (e.g., publication bias, time-lag bias, citation bias, and outcome bias) will be evaluated for all studies using a funnel plot. If less than 10 studies are included, reporting bias will be assessed qualitatively on the basis of characteristics of the included studies, instead of performing the funnel plots.

### Data synthesis and analysis

If there are sufficient trials with clinically similar populations and outcome measures, a meta-analysis of primary and secondary outcomes will be carried out using Review Manager 5 (Cochrane Community, London, UK.). We will use the Mantel-Haenszel method to pool the treatment effect of dichotomous data as risk ratio (RR) with 95% confidence intervals (CI). We will express continuous data as mean difference (MD) with 95% CI using an inverse variance method. We will report the result narratively if only one study contributes data for an outcome. We will combine the data in a meta-analysis when data for an outcome can be contributed by two or more trials. A fixed-effects model will be used to assess the results for heterogeneity. But if substantial heterogeneity is found, a random-effects model will be used instead.

We can only assess the statistical significance of the efficacy of a specific intervention using conventional meta-analysis. However, conventional meta-analysis cannot assess the amount of evidence that we used to estimate the intervention effect. To fill this vacancy, we will conduct trial sequential analysis to assess whether sufficient studies are identified to draw a firm conclusion on the efficacy of a specific intervention in cognitive rehabilitation after stroke.

### “Summary of findings” table

The following outcomes will be included in a summary of findings table (see Additional file 4): clinical changes in general cognitive function, clinical changes in domain-specific cognitive function (e.g., executive function, attention, memory, or perception), adverse effects (e.g., stroke, disability, or mortality), and QoL.

We will assess the certainty of the body of evidence using the Grading of Recommendations Assessment, Development, and Evaluation (GRADE) system. The following five GRADE considerations: risk of bias, consistency of effect, imprecision, indirectness, and risk of publication bias will be considered. We will conform to methods described in Chapter 14 of the *Cochrane Handbook for Systematic Reviews of Interventions* [51]. A narrative “summary of findings” table format will be created to present results if meta-analysis is not feasible.

### Subgroup analysis

The following trial characteristics were prespecified as of interest to explore reasons behind heterogeneity: ischemic stroke versus hemorrhagic stroke and pharmacological interventions versus nonpharmacological interventions. We will use the “test for subgroup differences” in Review Manager 5 (Cochrane Community, London, UK).

### Sensitivity analysis

If the heterogeneity is substantial, we will exclude studies with high risk of bias and undertake a sensitivity analysis of the primary outcomes.

## Discussion

This review might be the most comprehensive and updated review about cognitive rehabilitation interventions for patients with PSCI until now. We will provide an overview of the cognitive rehabilitation interventions, summarizing the current evidence and providing valuable information for trial design in the future. This review will compare the efficacy of different interventions and hopefully yield information about detailed information of the interventions, such as intervention delivery and intensity. This will help clinical practitioners in decision-making when choosing optimal interventions for patients with PSCI and support the development of clinical practice guidelines in the future.

However, our study has some limitations. The quality of trials is likely to be mixed, possibly with the majority of trials being small studies with a high risk of bias. Since we try to include any interventions designed to improve cognition in patients after stroke, the heterogeneity might be moderate or substantial, posing problems for evidence synthesis.

## Supplementary Information

**Additional file 1.** PRISMA-P 2015 Checklist.

**Additional file 2.** MEDLINE search strategy.

**Additional file 3.** Screening pilot-test form.

**Additional file 4.** Template for ‘Summary of findings’ table.

## Data Availability

Not applicable.

## Abbreviations

AHA/ASA: American Heart Association/American Stroke Association
CACR: Computer-assisted cognitive rehabilitation
CENTRAL: Cochrane Central Register of Controlled Trials
CI: Confidence intervals
CINAHL: Cumulative Index of Nursing and Allied Health Literature
CMBs: cerebral microbleeds
EMBASE: Excerpta medica database
GRADE: Grading of Recommendations, Assessment, Development and Evaluation
IMDoM: Informative missingness difference of means
IMOR: Informative missing odds ratio
MD: Mean differences
MMSE: Mini-Mental State Examination
MoCA: Montreal Cognitive Assessment
NIBS: Noninvasive brain stimulation
PRISMA: Preferred Reporting Items for Systematic Reviews and Meta-analysis
PSCI: Post-stroke cognitive impairment
RR: Risk ratios
rTMS: Repetitive transcranial magnetic stimulation
