# Supplementary material for: Cognitive rehabilitation interventions after stroke: protocol for a systematic review and meta-analysis of randomized controlled trials
Source: Syst Rev. 2021 Mar 4;10:66. doi: 10.1186/s13643-021-01607-7 (PMC7931553; doi:10.1186/s13643-021-01607-7)
Supplement: Supplementary file 2 — Additional file 2. MEDLINE search strategy. [file 13643_2021_1607_MOESM2_ESM.doc]

**Additional file 2**. MEDLINE search strategy.

| # No | Searches |
| --- | --- |
| *Part I: Stroke* | |
| 1 | Cerebrovascular Disorders/ or exp Basal Ganglia Cerebrovascular Disease/ or exp Brain Ischemia/ or Carotid Artery Diseases/ or Carotid Artery Thrombosis/ or Intracranial Arterial Diseases/ or Cerebral Arterial Diseases/ or exp "intracranial embolism and thrombosis"/ or exp Intracranial Hemorrhages/ or exp Stroke/ or exp Brain Infarction/ |
| 2 | (stroke$ or poststroke or cerebrovasc$ or brain vasc$ or cerebral vasc$ or cva$ or apoplex$ or SAH).tw. |
| 3 | ((brain$ or cerebr$ or cerebell$ or vertebrobasil$ or hemispher$ or intracran$ or intracerebral or infratentorial or supratentorial or middle cerebral artery or MCA or anterior circulation or posterior circulation or basilar artery or vertebral artery or basal gangli$) adj5 (isch?emi$ or infarct$ or thrombo$ or emboli$ or occlus$ or hypoxi$)).tw. |
| 4 | ((brain$ or cerebr$ or cerebell$ or intracerebral or intracran$ or parenchymal or intraparenchymal or intraventricular or infratentorial or supratentorial or basal gangli$ or putaminal or putamen or posterior fossa or hemispher$ or subarachnoid) adj5 (h?emorrhag$ or h?ematoma$ or bleed$)).tw. |
| 5 | 1 or 2 or 3 or 4 |
| *Part II: Cognition* | |
| 6 | exp Cognition/ or exp Cognition Disorders/ or exp Cognitive Dysfunction/ |
| 7 | ((cogniti$ or neurocognit$ or neuro-cognit$ or mental) adj5 (declin$ or impair$ or deficit$ or loss$ or deteriorat$ or degenerat$ or complain$ or disturb$ or disorder$ or concern$ or failure$ or dysfunct$ or difficult$ or problem$ or diminish$ or reduc$)).tw. |
| 8 | Neurobehavioral Manifestations/ or exp Confusion/ or exp Consciousness Disorders/ or exp Memory Disorders/ or exp Perceptual Disorders/ or Sensation Disorders/ or exp Hearing Disorders/ or exp Vision Disorders/ or exp Somatosensory Disorders/ or exp Apraxias/ |
| 9 | exp Attention/ or exp Memory/ or exp Perception/ or exp Sensation/ or exp Orientation/ or Spatial Navigation/ or Executive Function/ |
| 10 | (confusion$ or confused or agnosia$ or anosognosia$ or amnesia or inattention or distract$ or apraxia$ or dyspraxia$ or paracusis$).tw. |
| 11 | ((consciousness or awareness or attention$ or concentrat$ or alert$ or memor$ or recall or percept$ or hear$ or acoust$ or audit$ or visual$ or vision$ or ocular or orientat$ or spatial$ or hemispatial$ or hemi-spatial$ or visuospatial$ or visuopercept$ or visuo-spatial$ or visuo-percept$ or touch$ or tacti$ or somatosensat$ or somatic sensat$ or executive or dysexecutive) adj5 (declin$ or impair$ or deficit$ or loss$ or deteriorat$ or degenerat$ or complain$ or disturb$ or disorder$ or concern$ or failure$ or dysfunct$ or difficult$ or problem$ or diminish$ or reduc$ or neglect$ or disab$)).tw. |
| 12 | 6 or 7 or 8 or 9 or 10 or 11 |
| *Part III: Nonpharmacological interventions* | |
| 13 | exp Rehabilitation/ or exp Cognitive Behavioral Therapy/ or Cognitive Remediation/ or exp Complementary Therapies/ or exp Nutrition Therapy/ or exp Magnetic Field Therapy/ or exp Therapy, Computer-Assisted/ or exp Relaxation Therapy/ or Virtual Reality Exposure Therapy/ or exp Biofeedback, Psychology/ or exp Health Behavior/ or exp Health Education/ or Recovery of Function/ |
| 14 | exp Exercise/ or exp Leisure Activities/ or exp Recreation/ or exp Automobile Driving/ or exp Transportation/ or exp Language/ or exp "Diet, Food, and Nutrition"/ or exp Computer Systems/ or exp Software/ or exp Computer Simulation/ or Computer-Assisted Instruction/ or Music/ or exp "Task Performance and Analysis"/ or Acupuncture/ |
| 15 | ((cogniti$ or neurocognit$ or neuro-cognit$ or memor$ or mnemon$ or psycho$ or neuropsycholog$ or neuro-psycholog$ or behavio?r$ or neurobehavi?or$ or neuro-behavi?or$ or computer$ or compensat$ or relaxat$ or body-mind$ or mind-body$ or physical or art or occupant$) adj3 (training$ or retraining$ or intervent$ or therap$ or treatment$ or stimulat$ or educat$ or rehabilitat$ or neurorehabilitat$ or neuro-rehabilitat$ or psychotherap$ or psycho-therap$ or psychoeducat$ or psycho-educat$ or physiotherap$ or physio-therap$)).tw. |
| 16 | (tDCS or TMS or TBS or acupunct$ or electroacupunct$ or acupoint$ or feedback$ or biofeedback$ or neurofeedback$ or bio-feedback$ or neuro-feedback$ or NFT or exercis$ or danc$ or walking$ or running$ or stretching$ or driving$ or dressing$ or feeding$ or eating$ or bathing$ or washing$ or grooming$ or mobil$ or painting$ or drawing$ or gardening$ or mindfulness or Yoga or Tai-ji or "Tai Chi" or "Tai Ji" or Taiji or Qigong or "Qi Gong" or leisure or recreat$ or bibliotherap$ or reading$ or music$ or graph$ or game$ or virtual reality$ or VR or diet$ or food$1 or nutrit$ or n?utraceut$ or fruit$ or veget$ or salt$ or c?ffe$ or alcohol$ or smok$ or tobacco$ or supplement$ or fish oil or vitamin$ or probiotic$).tw. |
| 17 | 13 or 14 or 15 or 16 |
| *Part VI: Pharmacological interventions* | |
| 18 | exp Pharmacology/ or exp Drug Therapy/ or exp Pharmaceutical Preparations/ |
| 19 | exp Cholinesterase Inhibitors/ or Donepezil/ or Galantamine/ or Rivastigmine/ |
| 20 | (((acetylcholinesterase$ or cholinesterase$ or anticholinesterase$ or anti-cholinesterase$) adj3 (drug$ or agent$1 or inhibitor$)) or donepezil$ or galant?amin$ or lycoremine or nivalin$ or razadyne or reminyl or rivastigmine$ or exelon or huperzine A).tw. |
| 21 | Receptors, N-Methyl-D-Aspartate/ai [Antagonists & Inhibitors] |
| 22 | Excitatory Amino Acid Antagonists/ or Memantine/ |
| 23 | (((excitatory amino acid or EAA or glutamate or NMDA or "N-methyl-D-aspartate" or "N-methylaspartate") adj3 (antagonist$ or inhibitor$)) or memantin$ or namenda$ or axura$ or akatinol$ or ebixa$ or abixa$).tw. |
| 24 | Calcium Channel Blockers/ or Nimodipine/ or Piracetam/ or Nicergoline/ |
| 25 | ((calcium adj3 (block$ or antagonist$ or inhibitor$)) or vinpocetin$ or cavinton or kavinton or "ethyl apovincaminate" or periwinkle or butylphthalide or nimodipin$ or nymalize or nimotop or nymalize or nimotop or p?ra?etam or pyramem or nootrop or nootropil or nootropyl or nicergo$ or sermion or oxiracetam).tw. |
| 26 | Serotonin Uptake Inhibitors/ or Citalopram/ or Sertraline/ or Fluoxetine/ |
| 27 | (((serotonin or 5-HT or 5-hydroxytryptamine) adj5 (uptake or reuptake or re-uptake) adj5 inhibitor$) or SSRI$ or SNRI$ or citalopram$ or celexa or escitalopram or Lexapro or seropram or cipramil or sertralin$ or zoloft or lustral or fluoxetin$ or fluoxeren or fontex or ladose or sarafem or Prozac).tw. |
| 28 | (Neuroprotective Agents/ and exp Peptides/) or Nootropic Agents/ or Cytidine Diphosphate Choline/ |
| 29 | ((nootropic$ adj3 (drug$ or agent$1)) or c?tic?oline or CDP-choline or cerebrolysin$ or CERE or actovegin or cellex or brain polypeptide).tw. |
| 30 | exp Phosphodiesterase Inhibitors/ or Cilostazol/ or Pentoxifylline/ |
| 31 | ((phosphodiesterase adj3 (antagonist$ or inhibitor$)) or antiphosphodiesterases or cilostazol$ or pleta?l or pentoxif?lin$ or pentoxil or agapurin or oxpentifylline or trental).tw. |
| 32 | exp Methylphenidate/ or Modafinil/ |
| 33 | (methylphenidat$ or biphentin$ or centedrin$ or concerta$ or daytrana$ or dexmethylphenidat$ or equasym$ or focalin$ or medikinet$ or metadate$ or methylin$ or phenidyl$ or ritalin$ or rubifen or tsentedrin$ or quillivant or modafinil$ or armodafinil or modiodal or nuvigil or provigil or sparlo).tw. |
| 34 | Simvastatin/ or Pravastatin/ or Probucol/ |
| 35 | (pravastatin$ or eptastatin or vasten or lipemol or liplat or prareduct or mevalotin or pravachol or elisor or selektine or lipostat or pravacol or pravasin or bristacol or simvastatin$ or cholestat or colemin or labistatin or lipex or medipo or nivelipol or simovil or sinvacor or sivastin or statex or staticor or synvinolin or probucol$ or lurselle or panavir or biphenabid or lorelco or superlipid or lesterol or sinlestal).tw. |
| 36 | Guanfacine/ or Perindopril/ or Telmisartan/ |
| 37 | (guanfacin$ or estulic or tenex or p?rindopril$ or telmisartan or pritor or micardis).tw |
| 38 | Pioglitazone/ or Metformin/ |
| 39 | (pioglitazone or actos or glucophage or metformin$).tw. |
| 40 | exp Aspirin/ or exp Dipyridamole/ or Clopidogrel/ |
| 41 | (aspirin$ or acetyl salicylic acid$ or acetyl?salicylic acid$ or dipyridamol$ or persantin$ or Curant?l or kurantil or antistenocardin or asasantin or aggrenox or clopidogrel or iscover or plavix).tw |
| 42 | Plants, Medicinal/ or exp Plant Preparations/ or Ginkgo biloba/ or Salvia miltiorrhiza/ or (Provinols/ and Pomegranate/) or Spermidine/ |
| 43 | (((china or chinese) adj2 (medicin$ or herb$ or drug$ or traditional)) or TCM or TCHM or plant$1 or herb$).tw. |
| 44 | (((miltiorrhiza$ or chinese) adj3 salvia$) or ginkgo$ or gingko$ or gingkco$ or ginko$ or gincosan$ or bilobalid$ or tebonin$ or tanakan$ or rokan$ or danshen$ or dan shen$ or pomegranates or punicaceae or punica granatum or Shi Liu Fruit or spermidin$).tw. |
| 45 | 18 or 19 or 20 or 21 or 22 or 23 or 24 or 25 or 26 or 27 or 28 or 29 or 30 or 31 or 32 or 33 or 34 or 35 or 36 or 37 or 38 or 39 or 40 or 41 or 42 or 43 or 44 |
| *Part V: Study filter* | |
| 46 | randomized controlled trial.pt. |
| 47 | controlled clinical trial.pt. |
| 48 | randomized.ab. |
| 49 | placebo.ab. |
| 50 | clinical trials as topic.sh. |
| 51 | randomly.ab. |
| 52 | trial.ti. |
| 53 | 46 or 47 or 48 or 49 or 50 or 51 or 52 |
| 54 | exp animals/ not humans.sh. |
| 55 | 53 not 54 |
| *Part VI: Part III AND IV* | |
| 56 | 17 or 45 |
| *Part VII: Part I, II, V AND VI* | |
| 57 | 5 and 12 and 55 and 56 |
