# Supplementary material for: Cognitive rehabilitation interventions after stroke: protocol for a systematic review and meta-analysis of randomized controlled trials
Source: Syst Rev. 2021 Mar 4;10:66. doi: 10.1186/s13643-021-01607-7 (PMC7931553; doi:10.1186/s13643-021-01607-7)
Supplement: Supplementary file 3 — Additional file 3. Screening pilot-test form. [file 13643_2021_1607_MOESM3_ESM.doc]

**Additional file 3.** Screeninig pilot-test form

**Level 1 screening**

If you answer NO to any of these questions, the citation will be excluded. All other citations will be included in L2 screening.

1. Does the study include patients with post-stroke cognitive impairment?
   YES____ NO____ UNCLEAR____
2. Were the patients treated with pharmacological (e.g. anticholinergic therapy, antihypertensive therapy, antiplatedlet therapy or others) or nonpharmacological (e.g. conventional cognitive training, virtual reality, noninvasive brain stimulation or others) cognitive rehabilitation interventions?
   YES____ NO____ UNCLEAR____
3. Were the patients treated with one of the above cognitive rehabilitation interventions compared to each other?

YES____ NO____ UNCLEAR____

1. Is this a relevant study design (e.g., experimental, quasi-experimental, observational studies)?
   YES____ NO____ UNCLEAR____

**Level 2 screening**

If you answer NO to any of these questions, the citation/study will be excluded. All other full-text articles will be included.

1. Does the study include patients with post-stroke cognitive impairment diagnosed by any validated neuropsychological tests or experienced researchers?
   YES____ NO____ UNCLEAR____
2. Were the patients treated with pharmacological (e.g. anticholinergic therapy, antihypertensive therapy, antiplatelet therapy or others) or nonpharmacological (e.g. conventional cognitive training, virtual reality, noninvasive brain stimulation or others) cognitive rehabilitation interventions delivered alone?
   YES____ NO____ UNCLEAR____
3. Were the patients treated with one of the above treatments compared to each other?

YES____ NO____ UNCLEAR____

1. Does the study report at least one of our efficacy and safety outcomes of interest (e.g., any clinical changes in general or specific cognitive domain, adverse effects (stroke, disability or mortality) or quality of life)?
   YES____ NO____ UNCLEAR____
2. Is this a relevant study design (experimental, quasi-experimental, observational cohort, case-control or registry studies)?
   YES____ NO____ UNCLEAR____
