# Supplementary material for: Cognitive rehabilitation interventions after stroke: protocol for a systematic review and meta-analysis of randomized controlled trials
Source: Syst Rev. 2021 Mar 4;10:66. doi: 10.1186/s13643-021-01607-7 (PMC7931553; doi:10.1186/s13643-021-01607-7)
Supplement: Supplementary file 4 — Additional file 4. Template for ‘Summary of findings’ table. [file 13643_2021_1607_MOESM4_ESM.doc]

**Additional file 4. Template for ‘Summary of findings’ table**

| **Noninvasive brain stimulation compared with acupuncture for cognitive impairment rehabilitation** | | | | | | |
| --- | --- | --- | --- | --- | --- | --- |
| **Patient or population:** patients of either sex, any ethnicity, aged over 18, with the presence of cognitive impairment after being diagnosed with stroke  **Settings:** hospital  **Intervention:** noninvasive brain stimulation  **Comparison:** acupuncture | | | | | | |
| **Outcomes** | **Illustrative comparative risks* (95% CI)** | | **Relative effect (95% CI)** | **No. of participants (studies)** | **Quality of the evidence (GRADE)** | **Comments** |
|  | **Assumed risk** | **Corresponding risk** |  |  |  |  |
|  | **Control** | **Experimental** |  |  |  |  |
| **Clinical changes in general cognitive function** |  |  |  |  |  |  |
| **Clinical changes in executive function** |  |  |  |  |  |  |
| **Clinical changes in attention** |  |  |  |  |  |  |
| **Clinical changes in memory** |  |  |  |  |  |  |
| **Clinical changes in perception** |  |  |  |  |  |  |
| **Stroke** |  |  |  |  |  |  |
| **Disability** |  |  |  |  |  |  |
| **Mortality** |  |  |  |  |  |  |
| **QoL** |  |  |  |  |  |  |
| * The basis for the **assumed risk** (e.g. the median control group risk across studies) is provided in footnotes. The **corresponding risk** (and its 95% CI) is based on the assumed risk in the comparison group and the **relative effect** of the intervention (and its 95% CI). **CI**: confidence interval; **RR**: risk ratio; | | | | | | |
| **GRADE Working Group grades of evidence**  **High certainty:** we are very confident in the effect estimate; further research is very unlikely to change our confidence.  **Moderate certainty:** we are moderately confident in the effect estimate; further research is likely to impact our confidence.  **Low certainty:** we have limited confidence in the effect estimate; further research is very likely to impact our confidence.  **Very low certainty:** our confidence in the effect estimate is very little; | | | | | | |
